# Empowering lay carers to administer end-of-life medications: A crucial step in supporting home deaths

**DOI:** 10.1017/S1478951526102399

**Published:** 2026-05-15

**Authors:** Agus Setiawan, Andika Ari Saputra, Imelda Ratih Ayu, Arbin Janu Setiyowati, Nur Eva

**Affiliations:** 1 Faculty of Education, Universitas Persatuan Guru Republik Indonesia Semarang, Semarang, Indonesia; 2 Faculty of Education, Universitas Negeri Malang, Malang, Indonesia; 3 Faculty of Education, Universitas PGRI Palembang, Palembang, Indonesia; 4 Faculty of Psychology, Universitas Negeri Malang, Malang, Indonesia

Dear Editor,

We are writing in response to the insightful article “What can we learn from the accounts of lay carers administering end-of-life medications to a loved one at home? Exploring benefits, challenges and ways to empower patients and carers in the future” by Hendry et al. (2026). The study offers a deep exploration of the complex role that lay carers play in facilitating a home death by administering as-needed subcutaneous medications for breakthrough symptoms. While the article provides significant insight into the practice, we wish to address key aspects that can enhance the applicability of this intervention for both carers and healthcare professionals (HCPs).

The study powerfully underscores the *empowerment* experienced by carers who are trained to administer medication to their loved ones at the end of life. The desire to fulfill a dying person’s wish to die at home is a compelling reason for lay carers to embrace the responsibility of medication administration (Yang et al. 2024; Poolman et al. 2025). As highlighted, the emotional burden that accompanies caring for a loved one during their last days can be overwhelming, yet the ability to manage symptoms effectively offers comfort, reduces isolation, and enhances the dying process (Hendry et al. 2026). This aligns with previous findings that demonstrate how family members, through proper support and training, can contribute significantly to symptom management and reduce the need for emergency interventions (Wang et al. 2024).

However, while the benefits of lay carer administration are clear, there are critical challenges that must be acknowledged. Chief among them is the fear of making mistakes in symptom recognition and medication administration, which could inadvertently hasten death or cause harm (Yang et al. 2024). Carers in the study expressed concerns about the potential for error, particularly when unsure about recognizing breakthrough symptoms or using medical equipment correctly. These anxieties highlight the need for comprehensive, ongoing training and robust support systems to reassure carers and ensure patient safety.

A key recommendation we wish to reinforce is the importance of *timing and accessibility* in introducing the practice. As the study indicates, carers with patients who deteriorated more slowly felt better prepared, whereas those with rapid deterioration wished they had been approached earlier to discuss medication administration (Hendry et al. 2026). Given the unpredictability of the end-of-life phase, it is crucial that healthcare providers initiate discussions and training well in advance of any immediate need. In addition, addressing the *delays in symptom control* due to the time it takes HCPs to arrive at the home is vital. Empowering carers to act swiftly when symptoms break through can significantly improve the quality of care and alleviate patient distress, particularly in rural areas where delays are more pronounced (Salikhanov et al. 2023).

We also recognize the importance of *emotional support* for carers. While empowering carers with knowledge and practical skills is essential, it is equally important to provide them with emotional and psychological support throughout the process. The burden of caregiving, especially in the emotional context of administering medications to a loved one, can lead to stress, guilt, and anxiety (Bongelli et al. 2024). It is crucial that HCPs ensure carers feel confident and supported in their decision-making and provide regular check-ins to address any concerns they may have.

The findings of this study contribute significantly to the growing evidence supporting the role of lay carers in end-of-life care, emphasizing how *training and communication* can enable carers to provide compassionate, timely care at home. However, as we continue to explore and implement this practice, we must prioritize the development of training models that are adaptable to the needs of diverse carers, ensuring that all lay carers, regardless of their previous experience with medical tasks, are equipped to take on this responsibility safely.

In conclusion, Hendry et al. (2026) provide a valuable contribution to the understanding of lay carers’ experiences with medication administration at the end of life. We strongly support the broader implementation of this practice, but stress the importance of addressing the emotional, practical, and logistical challenges that carers face. By ensuring that all carers are adequately trained and supported, we can create a more effective, compassionate model of care that empowers families to fulfill their loved ones’ final wishes while minimizing distress and harm.
